# MiR-513b-5p represses autophagy during the malignant progression of hepatocellular carcinoma by targeting PIK3R3

**DOI:** 10.18632/aging.203135

**Published:** 2021-06-13

**Authors:** Wei Jin, Yilei Liang, Shuyou Li, Guoxiang Lin, Haiying Liang, Zhenni Zhang, Weiming Zhang, Rongjun Nie

**Affiliations:** 1 Department of Hepatobiliary Surgery, Affiliated Wuming Hospital, Guangxi Medical University, Nanning, Guangxi Province, China; 2 Department of Maxillofacial Surgery, Affiliated Wuming Hospital, Guangxi Medical University, Nanning, Guangxi Province, China; 3 Department of Oncology and Intervention, Affiliated Wuming Hospital, Guangxi Medical University, Nanning, Guangxi Province, China; 4 Department of Gynecology, Affiliated Wuming Hospital, Guangxi Medical University, Nanning, Guangxi Province, China

**Keywords:** hepatocellular carcinoma, autophagy, progression, miR-513b-5p, *PIK3R3*

## Abstract

Hepatocellular carcinoma (HCC) serves as a prevailing global malignancy with severe mortality and extremely unsatisfactory prognosis, in which autophagy is a fundamental process in liver cancer pathogenesis, but the mechanisms are poorly understood. MicroRNAs (miRNAs) serve as a type of well-recognized non-coding regulators and contribute to the modulation of liver cancer development, from the aspects of diagnosis, progression, and therapy. Here, we aimed to investigate the function of hsa_microRNA-513b-5p (miR-513b-5p) in regulating autophagy during HCC progression. Specifically, our data showed that miR-513b-5p mimic reduced the LC3-II and beclin1 expression but enhanced p62 expression in HCC cells. MiR-513b-5p repressed liver cancer cell proliferation, migration/invasion, and induced apoptosis *in vitro*. Crucially, miR-513b-5p attenuated tumor growth of liver cancer cells *in vivo*. In the mechanical investigation, we identified that *PIK3R3* mRNA 3′UTR was targeted by miR-513b-5p and miR-513b-5p suppressed *PIK3R3* expression. *PIK3R3* overexpression partly reversed miR-513b-5p-mediated autophagy, proliferation, and apoptosis of liver cancer cells. Consequently, we concluded that miR-513b-5p repressed autophagy during the malignant progression of HCC by targeting *PIK3R3*. MiR-513b-5p may be applied as a therapeutic target for HCC.

## INTRODUCTION

Liver cancer is a prevalent malignancy and the principal reason for tumor mortality globally, in which hepatocellular carcinoma (HCC) depicts 70–85% of the entire liver carcinoma weight [1]. Although there have been recent improvements, most HCC cases are identified at an advanced stage, resulting in bad outcomes and a considerable recurrence frequency [2]. Though previous investigations have explored the unusual expression of various proteins in HCC, the mechanism of HCC development is still mostly undiscovered [3]. Autophagy is a lysosome-related program and represents an intricate function during tumorigenesis [4]. When denied growth factors, nutrients, and oxygen, cancer cells sustain their durability by autophagy-associated degeneration of damaged organelles and misfolded proteins [5]. As the previous studies, autophagy is a crucial process during liver cancer development and a potential therapeutic target for liver cancer therapy (Allaire, 2019 #35; Huang, 2018 #34; Tang, 2019 #33), but the mechanisms are poorly understood.

MicroRNAs (miRNAs) are extensively expressed in many species, including viruses, plants, and animals [6, 7]. MiRNAs serve the non-coding, endogenous, and small regulators and negatively modulate the targeted genes' mRNA 3′-untranslated region (3′-UTR) by causing mRNAs degeneration suppressing protein translation [8–10]. Multiple miRNAs are reported to participate in the regulation of liver cancer [11, 12]. Meanwhile, miR-513b-5p has been reported to inhibit progression of embryonal carcinoma and lung cancer [13, 14]. Moreover, it has been uncovered that miR-511 reduces migration, invasion, and proliferation of HCC cells by targeting phosphoinositide-3-kinase regulatory subunit 3 [15]. MiR-132 represses migration, invasion, and proliferation of HCC cells through down-regulating *PIK3R3* [16]. *PIK3R3* acts as an oncogene of various cancers, containing glioma, lung cancer, and gastric cancer [17–19]. However, the impact of miR-513b-5p on *PIK3R3* in HCC is still unreported. As several miRNAs are involved in the modulation of autophagy in HCC and based on the crucial role of miR-513b-5p in cancer development, we selected miR-513b-5p as an example to evaluate its function in autophagy during liver cancer progression.

In the present study, we were interested in the miR-513b-5p function in the modulation of autophagy during liver cancer progression. We demonstrated that miR-513b-5p attenuated autophagy during the malignant progression of liver cancer by targeting PIK3R3.

## RESULTS

### MiR-513b-5p inhibits autophagy in liver cancer cells

**Figure 1:**
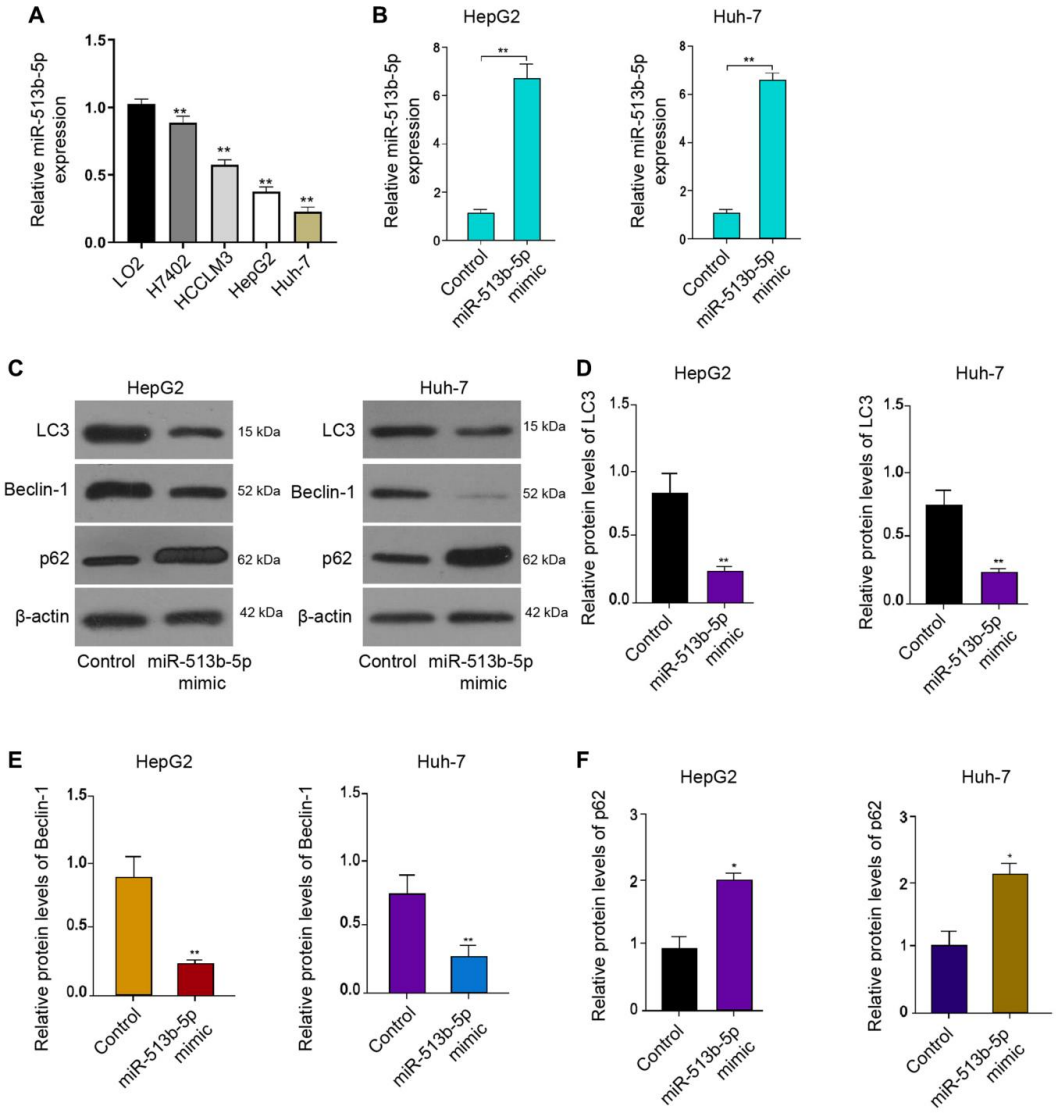
**MiR-513b-5p inhibits autophagy in liver cancer cells.** (**A**) The measurement of miR-513b-5p expression using qPCR. (**B**–**F**) The HepG2 and Huh-7 cells were treated with miR-513b-5p mimic. (**B**) The measurement of miR-513b-5p expression using qPCR. (**C**–**F**) The detection of LC3, beclin1, and p62 expression using Western blot analysis.

Firstly, we analyzed the expression of miR-513b-5p in the normal liver LO2 cells and liver cancer cells, including H7402, HCCLM3, HepG2 and Huh-7 cells. We observed that we miR-513b-5p was decreased in the liver cancer cells compared with the normal liver cells (Figure 1A). We then were interested in the function of miR-513b-5p in the regulation of autophagy in HCC, we analyzed the autophagy related markers, such as LC3, beclin1, and p62 in HCC cells. For this purpose, the liver cancer cell lines, including HepG2 and Huh-7 cells, were treated with miR-513b-5p and the expression of miR-513b-5p was enhanced in the cells (Figure 1B). Functionally, the treatment of miR-513b-5p mimic repressed the LC3-II and beclin1 expression but induced p62 expression in the HepG2, Huh-7, and HCCLM3 cells (Figure 1C–1F, Supplementary Figure 1). Taken together, these results indicate that miR-513b-5p inhibits autophagy in liver cancer cells.

### MiR-513b-5p represses liver cancer cell proliferation *in vitro*

**Figure 2:**
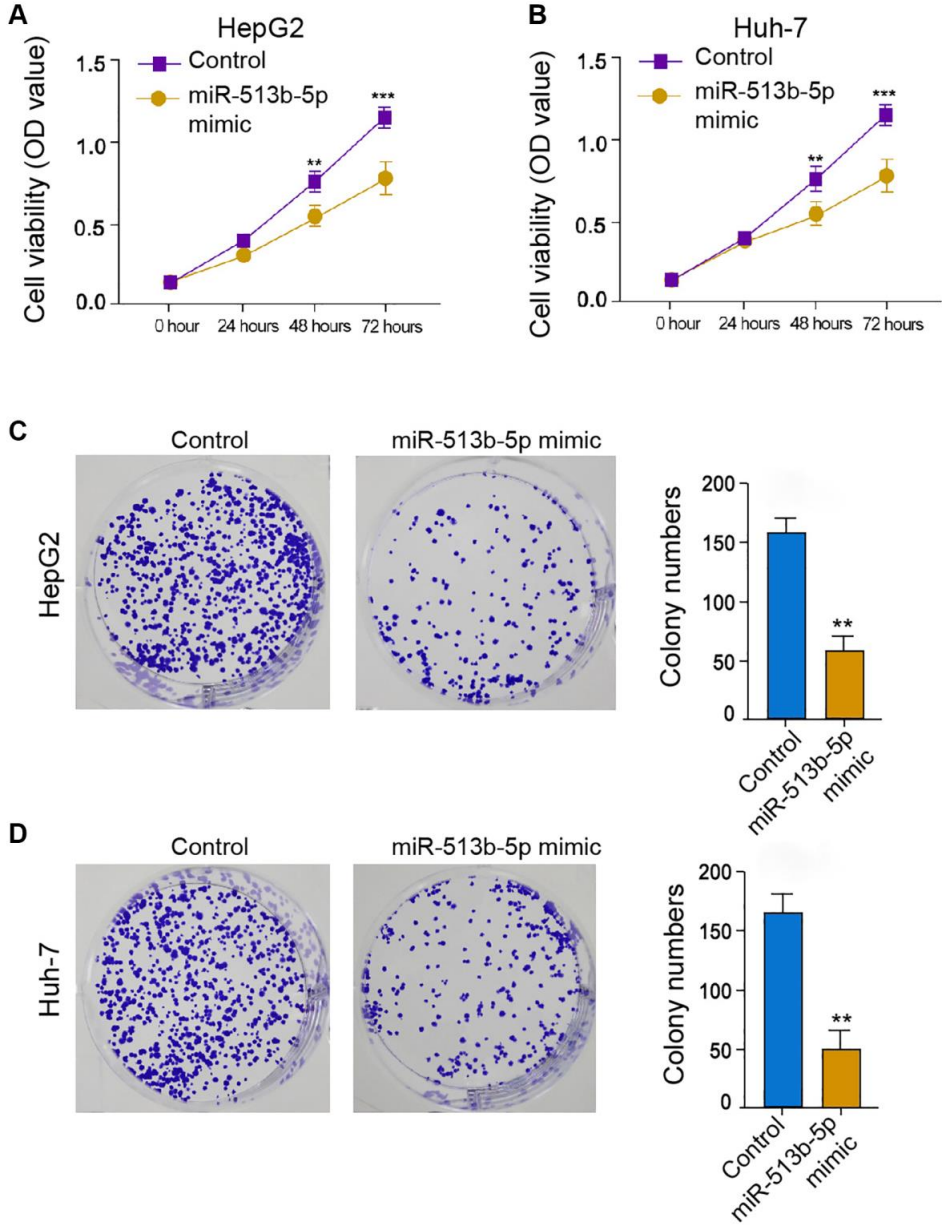
**MiR-513b-5p represses liver cancer cell proliferation *in vitro*.** (**A**–**D**) The HepG2 and Huh-7 cells were treated with miR-513b-5p mimic. (**A** and **B**) The analysis of cell proliferation using MTT assays. (**C** and **D**) The analysis of cell proliferation using colony formation assays.

We then further explored the effect of miR-513b-5p on HCC cell proliferation *in vitro*, to this end, we performed MTT assays and colony formation assays in the HepG2, Huh-7, and HCCLM3 cells. The treatment of miR-513b-5p suppressed the cell viability in the HepG2, Huh-7, and HCCLM3 cells (Figure 2A and 2B, Supplementary Figure 2A). Similarly, the colony formation ability of HepG2, Huh-7, and HCCLM3 cells was attenuated by miR-513b-5p (Figure 2C and 2D, Supplementary Figure 2B). Collectively, it suggests that miR-513b-5p represses liver cancer cell proliferation *in vitro*.

### MiR-513b-5p reduces tumor growth of liver cancer cells *in vivo*

**Figure 3:**
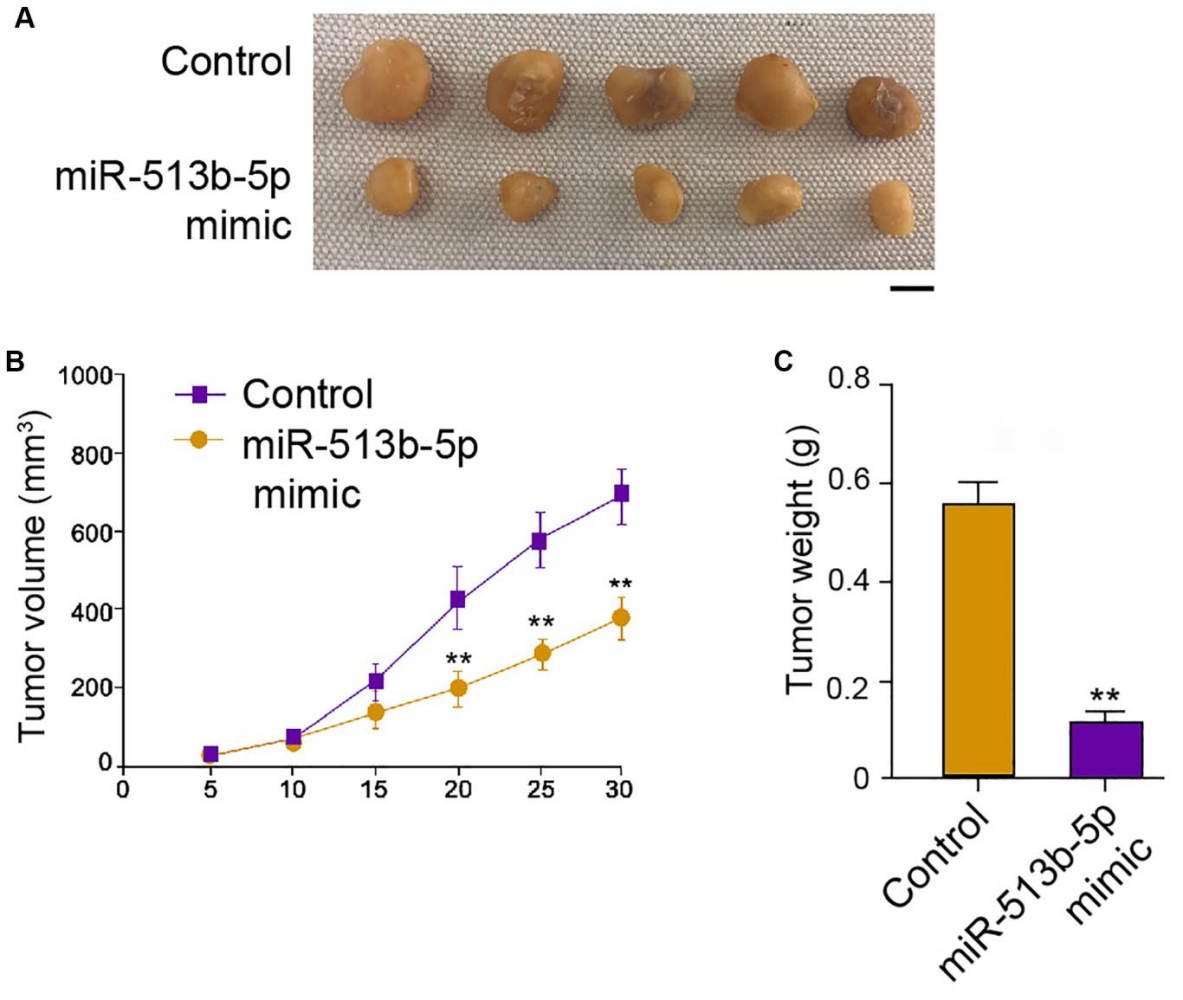
**MiR-513b-5p reduces tumor growth of liver cancer cells *in vivo*.** (**A**–**C**) The analysis of tumor growth of HepG2 cells treated with miR-513b-5p mimic using tumorigenicity assays in the nude mice. The tumor size (Scale bar = 10 mm), tumor weight, and tumor volume were shown.

We then further assessed the role of miR-513b-5p during tumor growth of HepG2 cells *in vivo*. Interestingly, the treatment of miR-513b-5p mimic remarkably alleviated the tumor growth phenotypes, including tumor size, tumor weight, and tumor volume (Figure 3A–3C). These data indicate that miR-513b-5p reduces tumor growth of liver cancer cells *in vivo*.

### MiR-513b-5p suppresses migration/invasion and enhances apoptosis of liver cancer cells *in vitro*

**Figure 4:**
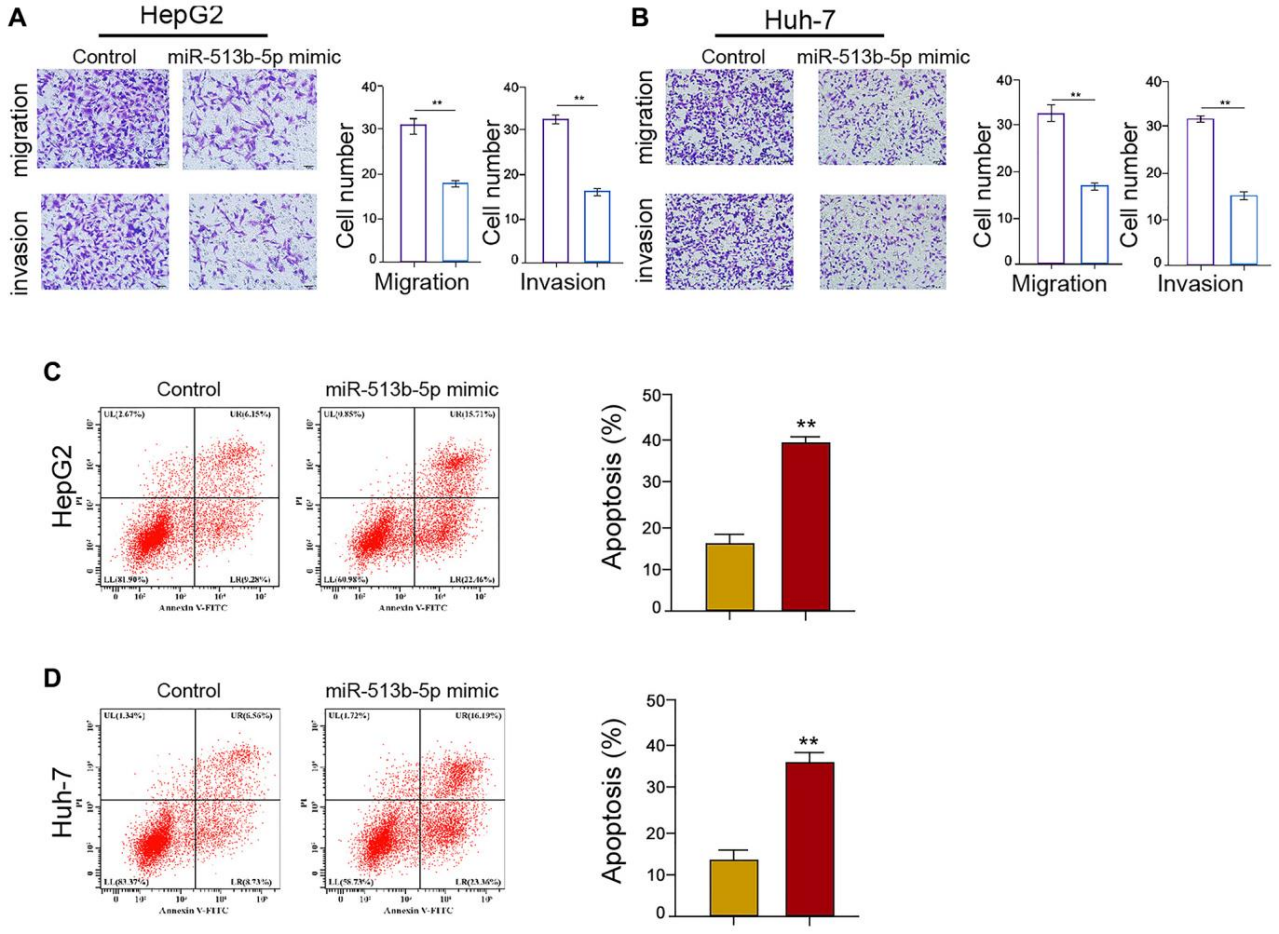
**MiR-513b-5p suppresses migration/invasion and enhances apoptosis of liver cancer cells *in vitro*.** (**A**–**D**) The HepG2 and Huh-7 cells were treated with miR-513b-5p mimic. (**A** and **B**) The analysis of cell migration/invasion using transwell assays. (**C** and **D**) The analysis of cell apoptosis using flow cytometry.

Given that the migration, invasion, and apoptosis are the crucial phenotype of cancer progression, we then concerned about the impact of miR-513b-5p on liver cancer cells migration/invasion and apoptosis *in vitro*. We found that miR-513b-5p mimic significantly restrained the migration/invasion capability of HepG2, Huh-7, and HCCLM3 cells (Figure 4A and 4B, Supplementary Figure 3A). Moreover, HepG2, Huh-7, and HCCLM3 cells apoptosis was induced in the miR-513b-5p mimic-treated cells (Figure 4C and 4D, Supplementary Figure 3B). Taken together, these data indicate that miR-513b-5p suppresses migration/invasion and enhances apoptosis of liver cancer cells *in vitro*.

### PIK3R3 is targeted by miR-513b-5p in liver cancer cells

**Figure 5:**
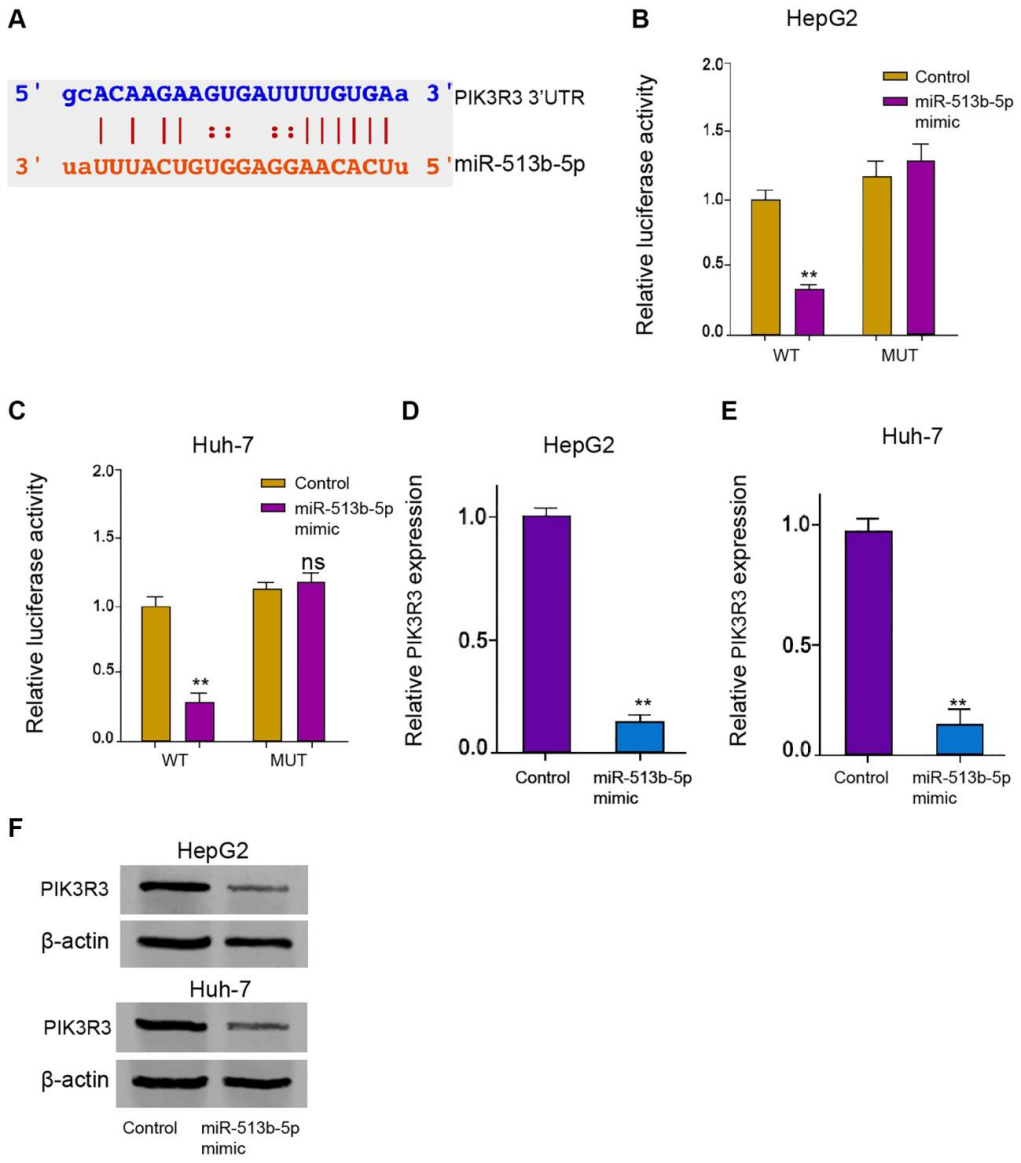
***PIK3R3* is targeted by miR-513b-5p in liver cancer cells.** (**A**) The interaction prediction analysis of miR-513b-5p with *PIK3R3* mRNA 3′UTR using ENCORI online database. (**B**–**E**) The HepG2 and Huh-7 cells were treated with miR-513b-5p mimic. (**B** and **C**) The analysis of luciferase activities using luciferase reporter gene assays. (**D** and **E**) The analysis of *PIK3R3* mRNA expression using qPCR. (**F**) The detection of *PIK3R3* expression using Western blot analysis.

Next, we tried to explore the potential mechanism underlying miR-513b-5p-mediated HCC progression. The predicted analysis demonstrated the potential binding of miR-513b-5p with *PIK3R3* mRNA 3′UTR (Figure 5A). MiR-513b-5p mimic remarkably repressed the luciferase activities of *PIK3R3* mRNA 3′UTR in the HepG2, Huh-7, and HCCLM3 cells (Figure 5B and 5C, Supplementary Figure 4). Both of the mRNA and protein levels of *PIK3R3* were down-regulated by miR-513b-5p mimic in the HepG2, Huh-7, and HCCLM3 cells (Figure 5D–5F, Supplementary Figure 3B). Collectively, it indicates that *PIK3R3* is targeted by miR-513b-5p in liver cancer cells.

### PIK3R3 is involved in miR-513b-5p-inhibited autophagy liver cancer cells

**Figure 6:**
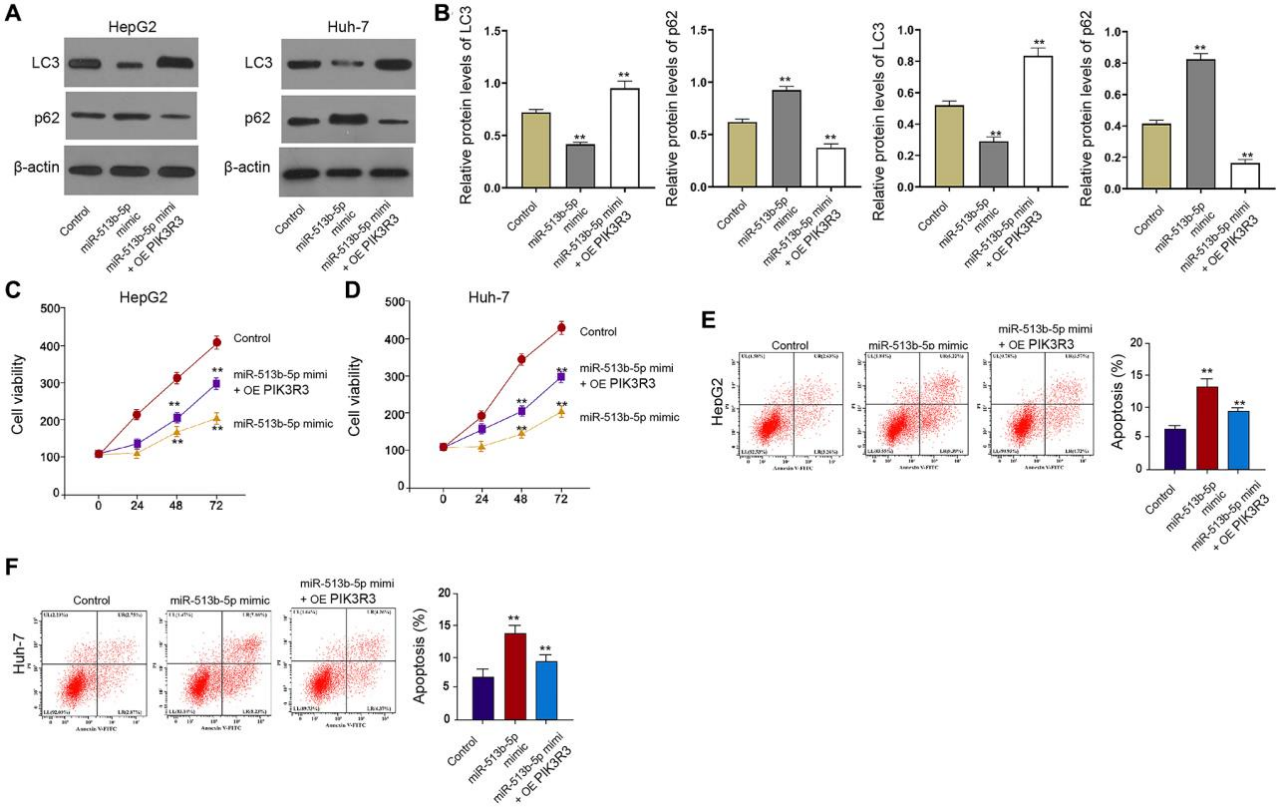
***PIK3R3* is involved in miR-513b-5p-inhibited autophagy liver cancer cells.** (**A**–**F**) The HepG2 and Huh-7 cells were treated with miR-513b-5p mimic and pcDNA3.1- *PIK3R3*. (**A** and **B**) The detection of LC3, beclin1, and p62 expression using Western blot analysis. (**C** and **D**) The analysis of cell proliferation using MTT assays. (**E** and **F**) The analysis of cell apoptosis using flow cytometry.

Furthermore, the treatment of miR-513b-5p mimic repressed the LC3-II expression but induced p62 expression in the HepG2 and Huh-7 cells, in which the *PIK3R3* overexpression partly reversed this effect in the cells (Figure 6A and 6B). Meanwhile, *PIK3R3* overexpression partly rescued the cell proliferation and blocked the cell apoptosis, which were mediated by miR-513b-5p mimic in the HepG2, Huh-7, and HCCLM3 cells (Figure 6C–6F, Supplementary Figure 5), implying that *PIK3R3* is involved in miR-513b-5p-inhibited autophagy liver cancer cells.

**Figure 7:**
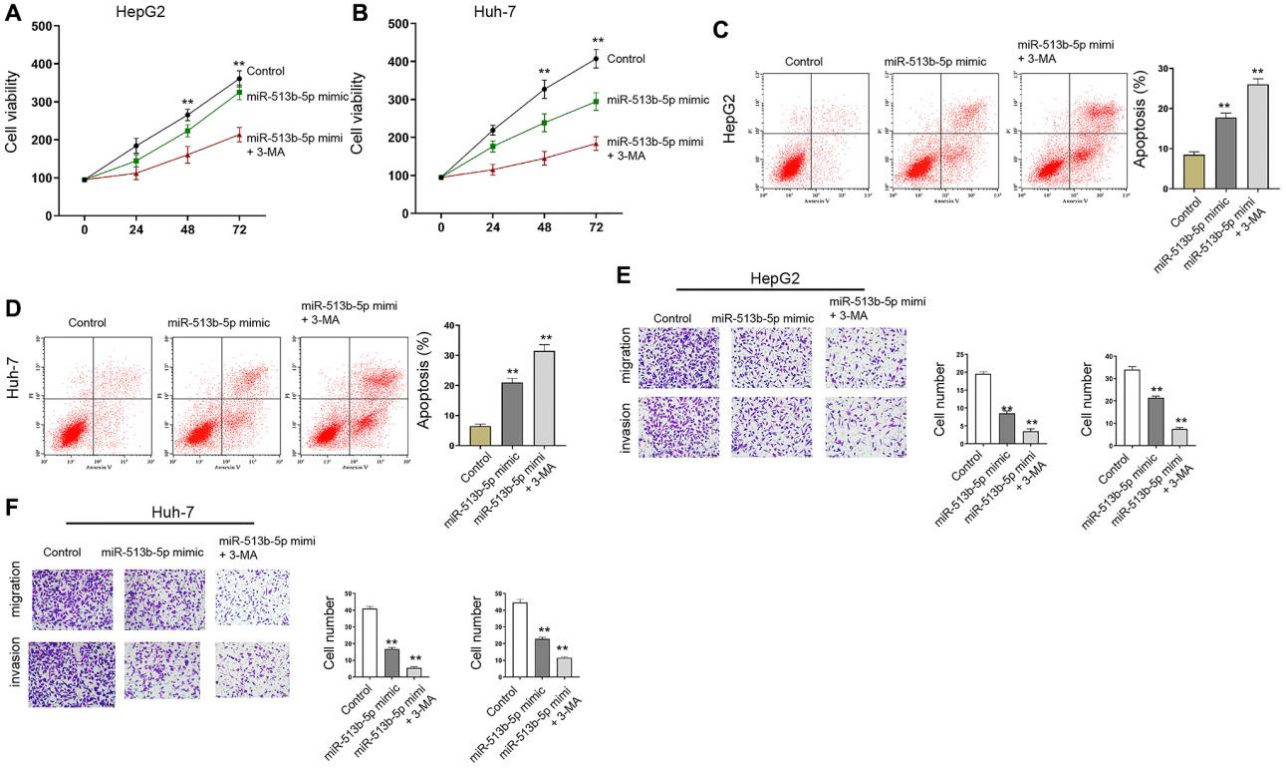
**Autophagy inhibitor 3-MA reverses miR-513b-5p-mediated liver cancer progression *in vitro*.** (**A**–**F**) The HepG2 and Huh-7 cells were treated with miR-513b-5p mimic and 3-MA (5mM). (**A** and **B**) The analysis of cell proliferation using MTT assays. (**C** and **D**) The analysis of cell apoptosis using flow cytometry. (**E** and **F**) The analysis of cell migration/invasion using transwell assays.

Moreover, autophagy inhibitor 3-MA enhanced miR-513b-5p mimic-promoted cell proliferation and miR-513b-5p mimic-inhibited cell apoptosis in the HepG2, Huh-7, and HCCLM3 cells (Figure 7, Supplementary Figure 6).

## DISCUSSION

It has been well-identified that autophagy is the critical cellular process and the regulators of autophagy are potential therapeutic targets for the liver cancer. The HGF/MET signaling controls autophagy and metabolism to regulate chemoresistance of liver cancer [20]. Autophagy regulates glycolytic metabolism by hexokinase 2 degradation in liver cancer [21]. Long noncoding RNA HULC enhances liver cancer progression *via* repressing PTEN through autophagy-mediated miR-15a [22]. Moreover, several miRNAs have been reported to regulate autophagy in liver cancer cells. MiR-638 promotes cell autophagy and apoptosis through repressing EZH2 in liver cancer [23]. MiR-223 enhancement attenuates doxorubicin-stimulated autophagy and relieves chemoresistance by targeting FOXO3 sin HCC cells [24]. In addition, it has been reported that miR-513b-5p enhances P53 expression by repressing IRF2 to decreases proliferation of testicular embryonal carcinoma cells [14]. MiR-513b-5p targets DUSP11 and is involved in AZIN1-AS1-promoted lung cancer progression [13]. Our data showed that miR-513b-5p decreased autophagy-related phenotypes in liver cancer cells. miR-513b-5p repressed proliferation and migration/invasion, and enhanced apoptosis *of* liver cancer cell *in vitro* and reduced tumor growth of liver cancer cells *in vivo*. These data indicate a novel function of miR-513b-5p in the autophagy modulation and liver cancer progression, providing important evidence of the role of miR-513b-5p in malignancies. In this study, we just evaluated the function of miR-513b-5p as a example in the modulation of autophagy in HCC, Whether or not have other miRNAs as well as miR-513b-5p to suppress autophagy in HCC progression and then synergy with miR-513b-5p should be explored in future investigations. Multiple factors may affect the outcome of miR-513b-5p-mediated autophagy and cancer progression. According to the previous study, several upstream factors, including long non-coding RNA FTX [25], long non-coding RNA UCA1 [26], long non-coding RNA LINC00861 [27], long non-coding RNA AZIN1-AS1 [13], circular RNA G004213 [28], and circular RNA circ_LARP4 [29], in the modulation of cancer progression, such as pancreatic cancer, osteosarcoma, cervical cancer, non-small cell lung cancer, and ovarian cancer. Whether these factors modulated miR-513b-5p-mediated autophagy in liver cancer should be validated in future investigations. Moreover, the miR-513b-5p mimic-enhanced p62 is associated with aggresome formation and maintain the survival of HCC. Thus, the miR-513b-5p is not sufficient to suppress the HCC relapse. More evidence should be constructed in future investigations to validate this issue.

Previous investigations have demonstrated the function of PIK3R3. It has been reported that miR-601 is a potential cancer inhibitor by repressing *PIK3R3* in HCC cells [30]. MiR-1287 represses the migration, invasion, and proliferation of HCC cells by targeting *PIK3R3* [31]. In our study, we identified that *PIK3R3* was targeted by miR-513b-5p in liver cancer cells and involved in miR-513b-5p-inhibited autophagy liver cancer cells. Our finding provides new knowledge of the mechanism involving *PIK3R3* of miR-513b-5p-mediated cancer progression. Meanwhile, *PIK3R3* may be just one of the downstream targets of *PIK3R3* in the modulation of cancer development, more potential factors response to miR-513b-5p-regulated cancer progression need to be explored in other investigations. In addition, some reported factors, including E2F5 [26], PTEN/AKT/mTOR signaling [27], DUSP11 [13], PRPF39 [28], and LARP4 [29], are involved in miR-513b-5p regulated cancer progression. The correlation of miR-513b-5p with these factors in the regulation of tumorigenesis and autophagy in liver cancer should be confirmed in future studies. There are still some limitations of this study. For example, we just used the miR-513b-5p mimic but not miR-513b-5p inhibitor to investigate the function of miR-513b-5p in HCC cells, the effect of miR-513b-5p inhibitor on autophagy in HCC cells needs to validate in further experiments. Meanwhile, we just evaluated the effect of miR-513b-5p on HCC cell growth *in vivo*, the function of miR-513b-5p/ *PIK3R3* needs to be confirmed in the model. Importantly, the clinical significance of miR-513b-5p in HCC should be assessed in clinical HCC samples. This study provides the new knowledge of the function of miR-513b-5p in regulation autophagy during HCC and the correlation of miR-513b-5p with *PIK3R3* in this process. The therapeutic agents or strategies of targeting miR-513b-5p/ *PIK3R3* axis should be developed and designed and may benefit the routine clinical practice in HCC. Meanwhile, it could promote the application of miR-513b-5p to be a potential drug in HCC.

We concluded that miR-513b-5p repressed autophagy during the malignant progression of HCC by targeting PIK3R3. MiR-513b-5p may be applied as a therapeutic target for HCC.

## MATERIALS AND METHODS

### Cell culture

The LO2, H7402, HCCLM3, HepG2, and Huh-7 cells were obtained in American Type Tissue Culture Collection. The cells were cultured in the DMEM (BI, USA) containing 0.1 mg/mL streptomycin (BI, USA), 100 units/mL penicillin (BI, USA), and 10% fetal bovine serum (BI, USA), at a condition of 37°C with 5% CO_2_. The lentiviral plasmids carrying miR-196a-5p mimic/inhibitor, and pcDNA3.1- *PIK3R3* were synthesized and obtained (Genscript, China). The autophagy inhibitor 3-Methyladenine (3-MA) were purchased from Selleck (USA).

### Quantitative reverse transcription-PCR (qRT-PCR)

The RNA was extracted from BC cells by using TriZol reagent (Thermo) after treatment, reverse transcribed to cDNA by using Super Script III kit (Invitrogen). Subsequently, the relative level was quantified by SYBR Premix kit (Takara, Japan), and normalized to GAPDH and 18 s. The results were calculated with 2^-△△Ct^ method. All primers were obtained from RiboBio (China).

### MTT assays

The cell viability was measured by MTT assays in the HepG2 and Huh-7 cells. Briefly, after the indicated treatment, about 2 × 10^4^ cells were put into 96 wells and cultured for 12 hours. After indicated treatment, the cells were added with the MTT solution (10 μL, 5 mg/mL) and cultured for an extra 4 hours. Discarded medium, and 150 μL DMSO was used to treat the wells. An ELISA browser was applied to analyze the absorbance at 570nm (Bio-Tek EL 800, USA).

### Western blot analysis

Total proteins were obtained from the mice tissues or cells with RIPA buffer (CST, USA). Protein concentrations were analyzed by applying the BCA Protein Quantification Kit (Abbkine, USA). Same concentration of protein was divided by SDS-PAGE (12% polyacrylamide gels), transferred to PVDF membranes (Millipore, USA) in the subsequent step. The membranes were hindered with 5% milk and hatched overnight at 4°C with the primary antibodies for *PIK3R3* (Abcam, USA), LC3 (Abcam, USA), beclin1 (Abcam, USA), p62 (Abcam, USA), and β-actin (Abcam, USA). Then, the corresponding second antibodies (Abcam, USA) were used for hatching the membranes 1 hour at room temperature, followed by the visualization by using an Odyssey CLx Infrared Imaging System.

### Colony formation assays

The HCC cells were transfected as the indication, digested and suspended as single cells, seeded into 6-well plates with 1000 cells in each well. The cells were placed in incubator for two weeks, and the medium was changed every three days till the visible clones formed. The formed clones were stained by 0.5% crystal violate resolved in methanol, captured and counted by a microscope (Leica, Germany).

### Tumorigenicity

The tumor growth of liver cancer cells *in vivo* was analyzed in nude mice of Balb/c (male, 4-week-old) (*n* = 5). About 1 × 10^7^ cells HepG2 cells treated with miR-513b-5p mimic A. After 5 days of injection, we measured tumor growth every 5 days. We sacrificed the mice after 30 days of injection, and tumors were scaled. The width and length of tumor, and the body weight of mice were measured at indicated time. Tumor volume was calculated by the formula: width (mm)^2^ × length (mm)/2. The mice were anesthetized to death when tumor size reached 1000 mm^3^, and the tumors were collected. Animal care and method procedures were authorized by the Animal Ethics Committee of Affiliated Wuming Hospital, Guangxi Medical University.

### Transwell assays

The migration and invasion ability of HCC cells were determined via using a transwell chamber (Corning, USA). To detect migration, HCC cells (1 × 10^5^ cells/well) transfected as the indication were seeded into the upper chambers with FBS-free medium, while the lower chambers were filled with complete DMEM medium. After 24 hours incubation, the membranes of upper chambers were fixed by 4% paraformaldehyde for 15 min, and stained by 0.5% crystal violet for 30 minutes. The migrated cells were photographed and counted. For cell invasion, the process was similar with that of migration experiment, only that the upper chambers were coated with Matrigel (BD Bioscience, USA).

### Analysis of cell apoptosis

For cell apoptosis detection, the apoptotic cells were stained with an FITC-Annexin V/PI detection kit (CST, USA). In brief, about 2 × 10^5^ HepG2 and Huh-7 cells were plated on 6-well dishes. And the cells were harvested, washed with PBS, then stained with FITC-Annexin V (5 μL) and PI (5 μL) for 10 minutes, respectively. The samples were then detected in a flow cytometry (BD Biosciences, USA).

### Luciferase reporter gene assay

The potential binding sites of miR-513b-5p and the 3′UTR region of *PIK3R3* were predicted by ENCORI website. The wild type sequences of the 3′UTR region of *PIK3R3* were cloned into the pmirGLO vectors (Promega, USA) to obtain the PIK3R3-WT. The site-specific mutated sequences of the 3′UTR of *PIK3R3* were inserted into pmirGLO vectors to obtain the PIK3R3-Mut. The cells were transfected with PIK3R3-WT and Mut along with miR-513b-5p mimic. After 24 hours incubation, the cells were lysed and the luciferase intensity was detected by a Dual Luciferase assay kit (Promega, USA). As control, the luciferase activities of Renilla were measured.

### Statistical analysis

Data were expressed as mean ± SD, and the statistical analysis was presented: ^*^P < 0.05, ^**^P < 0.01, ^***^P < 0.001, in which *P* < 0.05 were considered as statistically significant. The unpaired Student’s *t*-test and one-way ANOVA was used to compare the difference.

## Supplementary Materials

Supplementary Figures
