# Supplementary material for: MiR-513b-5p represses autophagy during the malignant progression of hepatocellular carcinoma by targeting PIK3R3
Source: Aging (Albany NY). 2021 Jun 13;13(12):16072–87. doi: 10.18632/aging.203135 (PMC8266330; doi:10.18632/aging.203135)
Supplement: Supplementary Figures [file aging-13-203135-s001.pdf]

SUPPLEMENTARY FIGURES

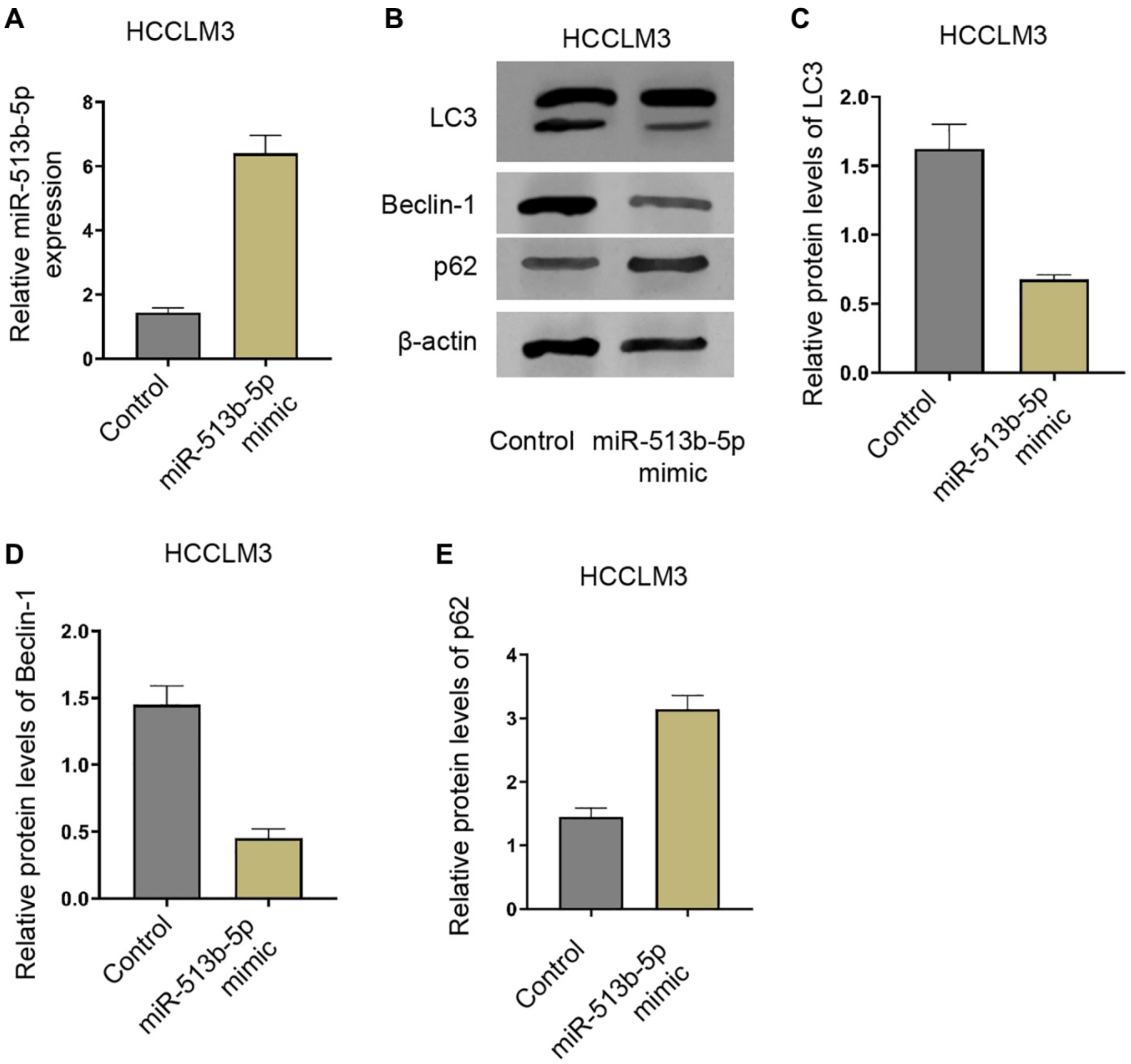

Supplementary Figure 1. MiR-513b-5p inhibits autophagy in liver cancer cells. (**A**–**E**) The HCCLM3 cells were treated with miR-513b-5p mimic. (**A**) The measurement of miR-513b-5p expression using qPCR. (**B**–**E**) The detection of LC3, beclin1, and p62 expression using Western blot analysis.

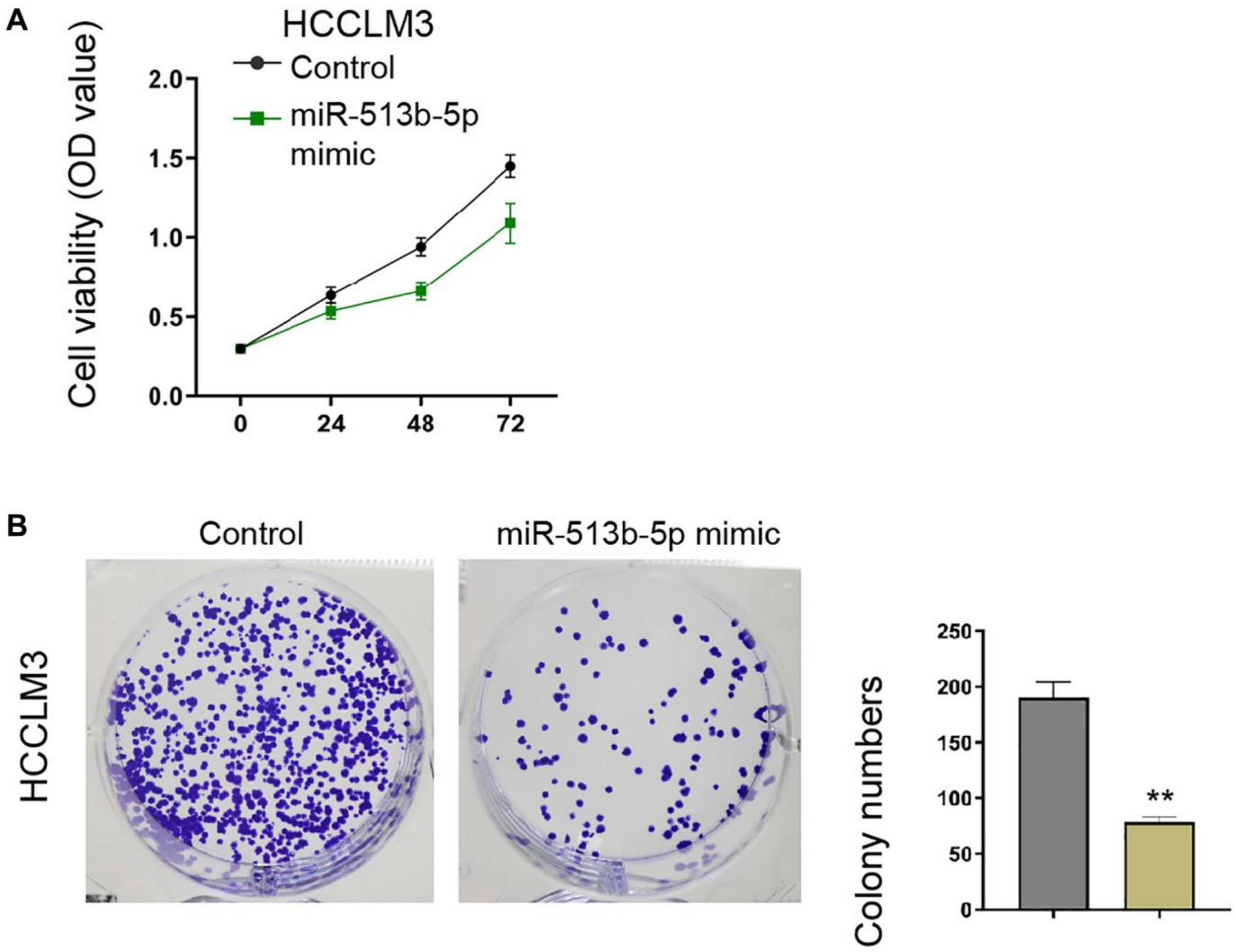

Supplementary Figure 2. MiR-513b-5p represses liver cancer cell proliferation *in vitro*. (**A** and **B**) The HCCLM3 cells were treated with miR-513b-5p mimic. (**A**) The analysis of cell proliferation using MTT assays. (**B**) The analysis of cell proliferation using colony formation assays.

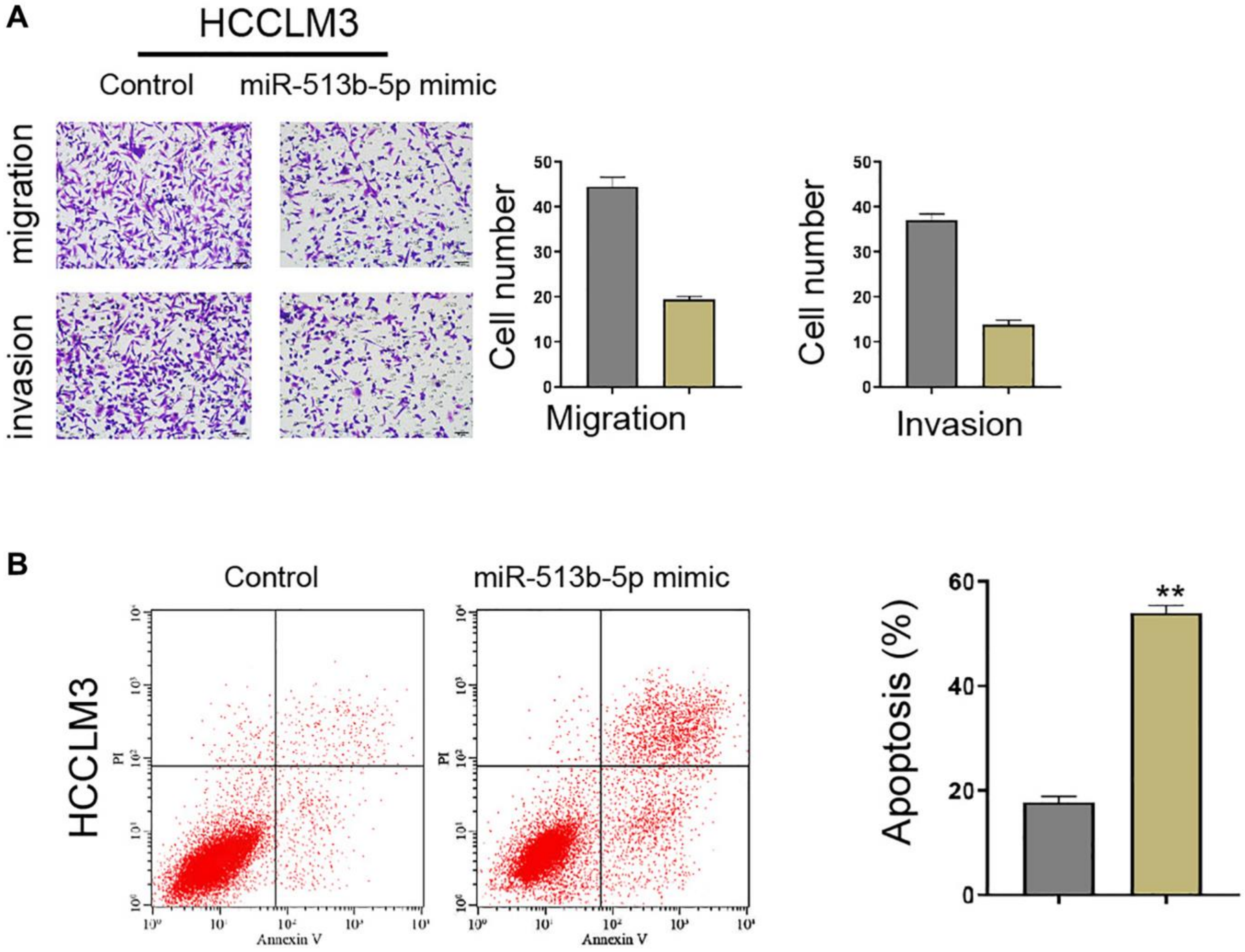

Supplementary Figure 3. MiR-513b-5p suppresses migration/invasion and enhances apoptosis of liver cancer cells *in vitro*. (**A** and **B**) The HCCLM3 cells were treated with miR-513b-5p mimic. (**A**) The analysis of cell migration/invasion using transwell assays. (**B**) The analysis of cell apoptosis using flow cytometry.

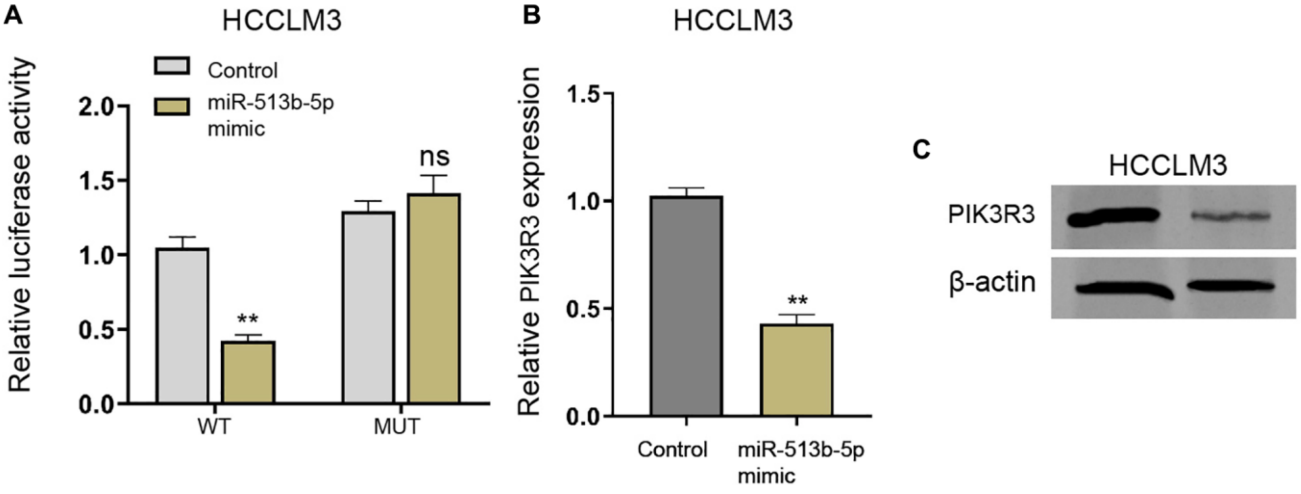

Supplementary Figure 4. *PIK3R3* is targeted by miR-513b-5p in liver cancer cells. (**A**–**C**) The HCCLM3 cells were treated with miR-513b-5p mimic. (**A**) The analysis of luciferase activities using luciferase reporter gene assays. (**B**) The analysis of *PIK3R3* mRNA expression using qPCR. (**C**) The detection of *PIK3R3* expression using Western blot analysis.

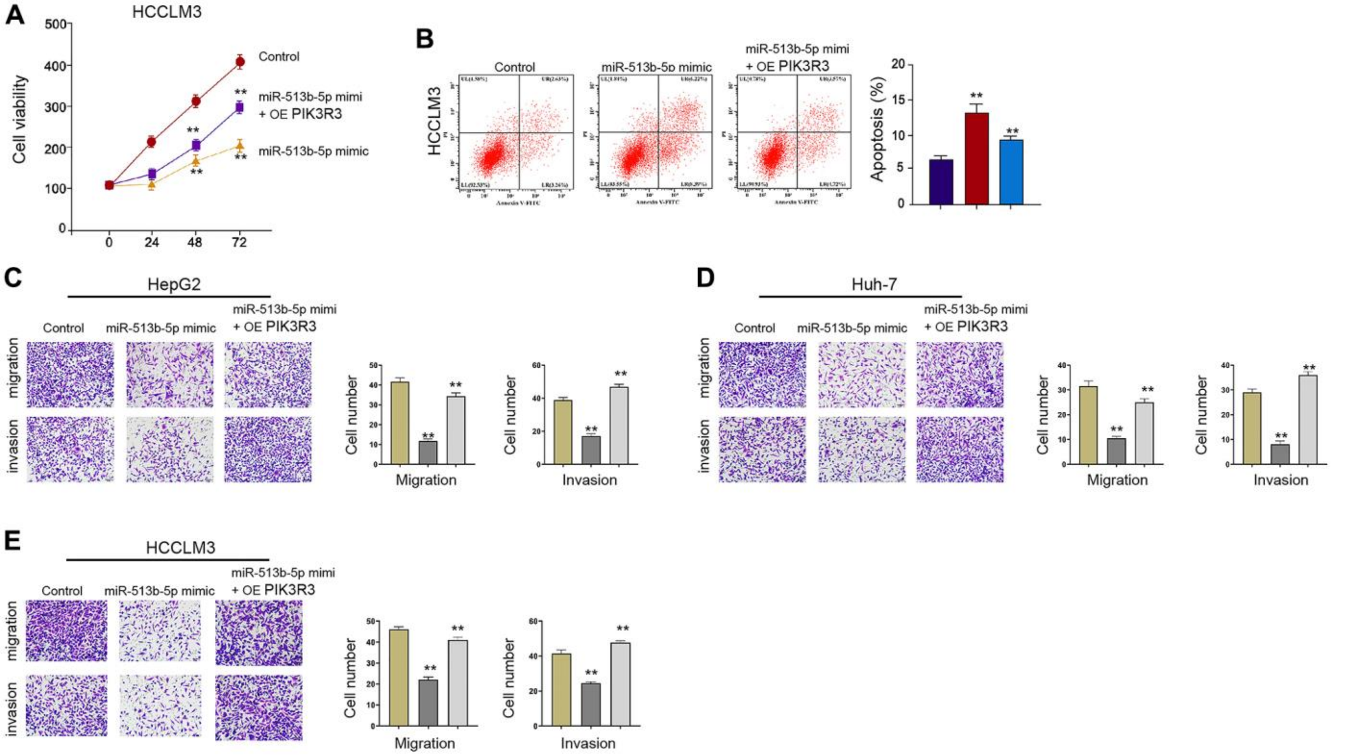

Supplementary Figure 5. *PIK3R3* is involved in miR-513b-5p-inhibited progression of liver cancer *in vitro*. (**A** and **B**) The HCCLM3 cells were treated with miR-513b-5p mimic and pcDNA3.1- *PIK3R3*. (**B**) The analysis of cell apoptosis using flow cytometry. (**C** and **D**) The HepG2 and Huh-7 cells were treated with miR-513b-5p mimic and pcDNA3.1- *PIK3R3*. The analysis of cell migration/invasion using transwell assays. (**E**) The HCCLM3 cells were treated with miR-513b-5p mimic and pcDNA3.1- *PIK3R3*. The analysis of cell migration/invasion using transwell assays.

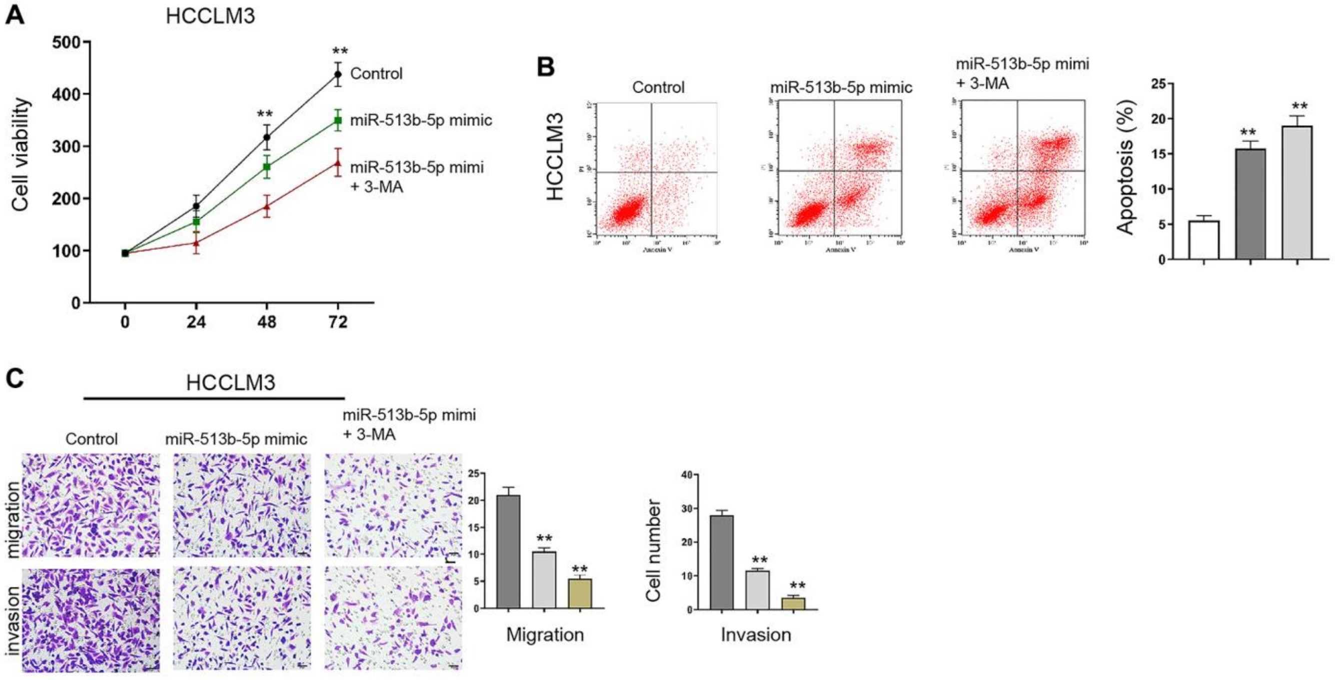

Supplementary Figure 6. Autophagy inhibitor 3-MA reverses miR-513b-5p-mediated liver cancer progression *in vitro*. (**A**–**C**) The HCCLM3 cells were treated with miR-513b-5p mimic and 3-MA (5 mM). (**A**) The analysis of cell proliferation using MTT assays. (**B**) The analysis of cell apoptosis using flow cytometry. (**C**) The analysis of cell migration/invasion using transwell assays.
